# Cyclic Gas Dissolution Foaming as an Approach for Simultaneously Reducing Cell Size and Relative Density in Nanocellular PMMA

**DOI:** 10.3390/polym13142383

**Published:** 2021-07-20

**Authors:** Judith Martín-de León, Victoria Bernardo, Miguel Ángel Rodriguez-Perez

**Affiliations:** 1 Cellular Materials Laboratory (CellMat), Condensed Matter Physics Department, University of Valladolid, 47011 Valladolid, Spain; marrod@fmc.uva.es; 2 CellMat Technologies S.L. Paseo de Belén 9A, 47011 Valladolid, Spain; v.bernardo@cellmattechnologies.com

**Keywords:** low-density nanocellular foam, gas dissolution foaming, PMMA, advanced cellular material

## Abstract

A new approach to produce nanocellular polymers combining small cell sizes with low relative densities is presented herein. This production method, based on gas dissolution foaming, consists of performing a double saturation and foaming cycle. Thus, nanocellular polymethylmethacrylate (PMMA) has been produced through a first saturation at different saturation conditions (6, 10, and 20 MPa and −32 °C), at constant foaming conditions (60 °C for 1 min). Then, the nanocellular PMMAs obtained from the previous step were again saturated at different saturation conditions, 10 MPa 24 °C, 31 MPa 24 °C, 35 MPa 22 °C, and 6 MPa −15 °C and foamed at different temperatures (40, 80 and 100 °C) for 1 min. This new approach allows the cells created in the first saturation and foaming cycle to further grow in the second cycle. This fact permits producing nanocellular polymethylmethacrylate sheets combining, for the first time in the literature, cell sizes of 24 nm with relative densities of 0.3.

## 1. Introduction

Nanocellular materials have aroused great attention due to a combination of outstanding properties, such as low thermal conductivity, improved mechanical properties, the possibility of retaining solid polymer transparency after the foaming process, among others [1,2,3,4]. Industries such as the automotive one or the building sector could improve their current designs by using these materials. However, so as to take advantage of properties, such as the thermal insulation, it is mandatory to reduce the density of the cellular material in addition to the cell size [5,6,7].

Since their discovery, nanocellular materials have been produced using different polymers and through different approaches [1]. The most common production route is the gas dissolution foaming process using both homogeneous and heterogeneous nucleation mechanisms. Those efforts have led to the production of unique cellular structures [4,8,9]. However, previous studies have demonstrated that there are some limitations regarding the production of these materials. In fact, it is still difficult to produce materials with very small cell sizes and low relative densities [1,10,11].

Polymethylmethacrylate (PMMA) is one of the most common polymers selected for the production of nanocellular polymers. This is due to its great affinity for CO_2,_ leading to high solubilities [10,12,13]. According to the classical nucleation theory, maximization of the solubility leads to an increase in the number of nucleation points resulting in an easy way to produce cells in the nanometric range by using a homogeneous nucleation approach. Particularly for this polymer, some of the most promising values when the materials are produced by the homogeneous nucleation approach have been presented by Yeh et al. [14] with cell sizes of 37 nm combined with a relative density of 0.25. However, the reported values were achieved for pellets and not for plates, and therefore the relative density values reported included the solid skin and the transition region with microcells and not only the nanocellular region (these two areas, skin and transition region, are difficult to remove in small pellets). Regarding nanocellular PMMA plates, the better values obtained through homogeneous nucleation have been presented by Martín-de León et al. with a cell size of 74 nm combined with a relative density of 0.24, (values obtained in the nanocellular area of these samples) [15]. The use of a copolymer (PMMA-co-EMA) allowed Costeux et al. to produce materials with cell sizes of 80 nm with relative densities of 0.17 (values obtained considering skin and transition region) for this particular polymer [16,17,18]. Kumar et al. presented very interesting materials with relative densities of 0.3 and cell sizes of 50 nm, or relative densities of 0.14 with cell sizes of 235 nm, being the reported relative density values with solid skin and transition region [19].

Regarding heterogeneous nucleation, some interesting results have also been obtained. Pinto et al. produced nanocellular PMMA by adding MAM (block copolymer poly(methyl methacrylate)-poly(butyl acrylate)-poly(methyl methacrylate) with cell sizes around 250 nm and relative densities around 0.5 [20]. Bernardo et al. reported cell sizes from 200 nm to 300 nm and relative densities of the nanocellular region from 0.23 to 0.47 by adding MAM as nucleating agent [21]. Wang et al. presented cell sizes of 150 nm with 0.17 of relative density by using TPU as nucleating agent. In this case, the used samples present a cylindrical shape of 5 mm in diameter and 50 mm in length. Density measurements do not include solid skin but they do include the transition region contributing to reduction of the density values [22]. Costeux et al. reported values of 120 nm-0.15, 99 nm-0.16 and 65 nm-0.26 of cell size-relative density (values including solid skin and transition region) using polyhedral oligomeric silsesquioxane (POSS) as the second phase. However, those works present the main drawback of the necessity of adding this second phase, leading to a more complex process [23].

On the other hand, when searching for the minimum cell size obtained with this polymer, the smaller values are presented by Martín-de León et al. [13]. In this work, cell sizes below 50 nm were reported with 39, 24, and 14 nm of cell size combined with relative densities of 0.38, 0.43, and 0.43, respectively, values obtained after removing the skin and transition region.

In summary, plates of nanocellular materials with minimum cell sizes (smaller than 40 nm) have never been reported to have densities below 0.4. While the minimum relative densities reported around 0.15 correspond to materials with cell sizes not smaller than 120 nm.

A new approach for producing nanocellular materials combining small cell sizes with low relative densities is proposed in the present work. This method proposes a cyclic gas dissolution foaming process allowing the production of materials not achievable using the common gas dissolution foaming process. Thus, nanocellular materials have been produced by means of a double saturation process leading to cell sizes as small as 24 nm (very close to the minimum value obtained so far) combined with relative densities of 0.3, a combination of values never reported before.

The influence of the production parameters, for both the first and the second saturation-foaming cycles, on the final cellular structure has been analyzed.

## 2. Materials and Methods

### 2.1. Materials

Polymethylmethacrylate (PMMA) is the material used in this work. V825T grade has been supplied in the form of pellets from ALTUGLAS^®^ International (Colombes, France).

The density of this material is 1.19 g/cm^3^ (measured at 23 °C and 50% HR), its molecular weight measured through gel permeation chromatography is M_n_ = 43 kg/mol, and M_w_ = 83 kg/mol and its glass transition temperature ($T_{g}$) is 114 °C (measured through differential scanning calorimetry). 

The used gas for the gas dissolution foaming experiments is CO_2_.

### 2.2. Samples Production

#### 2.2.1. Precursors Production

Solid sheets 4 mm in thickness have been compression molded through a cold/hot plate press (Remtex (Barcelona, Spain). 

The as-received pellets were first dried overnight at 70 °C under vacuum. The dry pellets were introduced in a 4 mm thickness mold. The mold was first heated in the hot plates at 250 °C for 9 min without applying pressure. Afterward, a pressure of 42 MPa was applied for one additional minute by keeping the temperature at 250 °C. Finally, the material is cooled down in the cold plates at room temperature under the same pressure. 

The obtained solid sheet is mechanized to 20 × 20 × 4 mm^3^ samples for the foaming experiments.

#### 2.2.2. Cellular Materials Production

A cyclic process based on the gas dissolution foaming technique has been used for producing the nanocellular materials of this work [24]. 

Gas dissolution foaming comprises three steps: saturation, depressurization, and foaming. During saturation, the polymer is introduced in a pressure vessel under certain gas pressure and temperature, parameters known as saturation pressure ($P_{sat}$) and saturation temperature ($T_{sat}$). The gas, normally CO_2_, diffuses inside the polymer until the sample is fully saturated. The gas diffusion inside the polymer implies the reduction of the glass transition temperature ($T_{g}$) down to the effective glass transition temperature $T_{g_{eff}}$. After saturation time $\left(t_{sat}\right)$, the gas is fast released in the depressurization step at a specific depressurization velocity $\left(v_{des}\right)$. This fast pressure drop results in a thermodynamic instability that creates nucleation points inside the polymer. Finally, in the foaming step, the polymer is introduced in a thermal bath at a foaming temperature $\left(T_{f}\right)$ higher than the effective glass transition temperature during the foaming time $\left(t_{f}\right)$. At these conditions, the nucleation points evolve into cells. 

**Figure 1:**
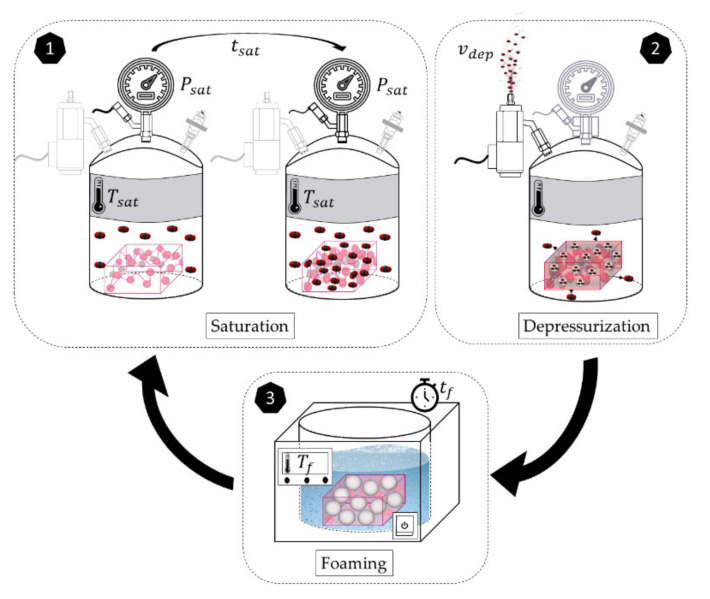
Scheme of the cyclic gas dissolution foaming process proposed in this work.

In this work, a cyclic process illustrated in Figure 1 is proposed. Once the cellular structure is produced and the CO_2_ of the first cycle has completely diffused out of the sample, a new gas dissolution foaming cycle is applied to the already foamed polymer. That means the produced nanocellular polymer in the first cycle is again saturated, depressurized, and foamed.

The described experiments have been carried out in a pressure vessel (model PARR 4681) provided by Parr Instrument Company (Moline, IL, USA). The pressure is controlled through a pressure pump (model SFT-10) supplied by Supercritical Fluid Technologies Inc. (Newark, DE, USA). Additionally, a clamp heater connected to a temperature controller CAL 3000 is used to adjust the temperature of the system. Finally, an electrovalve with K_v_ = 1.1 L/min allows fast release of the pressure.

A collection of experiments was performed to evaluate the effect of process conditions on the final cellular structure. The influence of the parameters in the first and second cycle is studied as it is detailed explained in Section 3.1.

### 2.3. Characterization Techniques

#### 2.3.1. Density

The density of the solid materials (ρ_s_) has been measured through gas pycnometry (Mod. AccuPyc II 1340, Micromeritics, Norcross, GA, USA). The density of the cellular materials $\left(\rho_{f}\right)$ has been determined through a density determination kit of an AT261 Mettler-Toledo balance, considering the water displacement method, based on Archimedes’ principle. 

Relative density ($\rho_{r})\;$ is defined as the fraction between the cellular material density and the solid one $\left(\rho_{r}=\rho_{f}/\rho_{s}\right)$. Cellular materials were polished, removing the solid skin and transition region before measuring the relative density. Polishing was done by means of a polisher model LaboPOl2-LaboForce 3 supplied by Struers. At least 200 µm were removed on each side making sure to remove all the thickness comprising solid skin and transition region. Therefore, all the data reported in this paper correspond to the homogeneous nanocellular region of the samples.

#### 2.3.2. Solubility

Solubility is defined as the percentage weight increase in the sample due to gas sorption. To determine solubility, the mass of the sample when it is fully saturated is needed. Since depressurization, the gas is diffusing out of the sample. To take this into account the mass loss vs. time was registered with a Mettler-Toledo balance. The mass of the sample when it is fully saturated is obtained by extrapolating to zero desorption time the measured mass [25].

#### 2.3.3. Scanning Electron Microscopy

The cellular structure of each cellular material was examined through scanning electron microscopy (ESEM, Model QUANTA 200 FEG, Hillsboro, OR, USA). The area analyzed is in the inert part of the sample, i.e., far from the transition layer or skin.

Before the visualization, samples were fractured under liquid nitrogen to preserve the cellular structure as produced. Afterward, the visualization surface was coated with gold with a sputter coater (model SDC 005, Balzers Union, Balzers, Liechtenstein).

The obtained micrographs have been analyzed with a software-based on ImageJ/FIJI. Different parameters have been measured to characterize the cellular structure. Average cell size (ϕ) has been measured as the mean value of the average diameter of more than 200 cells per sample, cell nucleation density (N_0_) was determined through Kumar’s method [24]. Finally, the standard deviation of the cell size distribution divided by the average cell size (SD/ϕ) has been also evaluated. 

## 3. Results

### 3.1. Example of the Nanocellular Materials Obtained When a Double Cycle Is Carried Out

**Figure 2:**
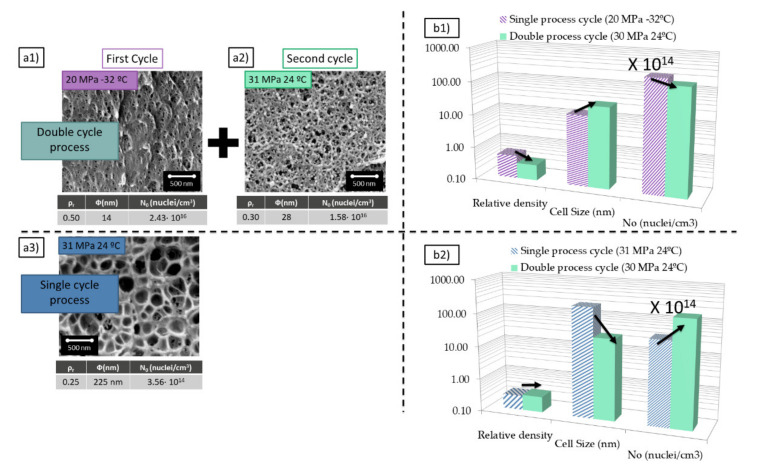
(**a**) Comparison between the cellular structures obtained after a first cycle at 20 MPa −32 °C and foamed at 60 °C for 1 min (**a1**) and a second cycle at 31 MPa, 24 °C and foamed at 80 °C for 1 min (**a2**) and the cellular structure obtained in a single cycle at saturation conditions of 31 MPa and 24 °C with foaming at 100 °C for 1 min (**a3**). (**b1**) Comparison between relative density, cell size, and cell nucleation density between (**a1**) and (**a2**). (**b2**) Comparison between relative density, cell size, and cell nucleation density between (**a3**) and (**a2**).

The PMMA grade used in this study shows cellular structures as those shown in the bottom of Figure 2a.

This material presents cell sizes around 200 nm and relative densities of 0.25 when it is saturated at 31 MPa and 24 °C and foamed at 100 °C for 1 min. These conditions are the ones minimizing the density for this PMMA grade when cell sizes are in the range of 200 nm. The cell nucleation density for these conditions is in the range of 3 × 10^14^ cells/cm^3^. The process parameters of this experiment are very the most appropriate to reach low densities at medium cell sizes.

On the other hand, when this material is saturated at 20 MPa and −32 °C presents cell sizes around 14 nm combined with a relative density of 0.5 (foaming at 60 °C for 1 min) (Figure 2a1). Once again these are the optimum conditions to reduce the density when the cell size is in the range of 14 nm. The cell nucleation density for these particular conditions is in the range of 2.4 × 10^16^ cells/cm^3^, i.e., two orders of magnitude higher than in the previous conditions. These process parameters minimize cell size but does not allow further reduction of density.

The introduction of a gas dissolution foaming consisting of two cycles that combine the two previous saturation cycles allows obtaining a cellular structure with a combination of characteristics that are not achieved through a single cycle process (Figure 2a3). Nanocellular PMMA with 28 nm of cell size and a relative density of 0.3 has been produced through a first saturation at 20 MPa and −32 °C and foaming at 60 °C for 1 min and a second saturation at 31 MPa and 24 °C and foaming at 80 °C for 1 min (Figure 2a2). For these conditions, the cell nucleation density is also very high, with values of 1.6 × 10^16^ cells/cm^3^. Therefore, using this new approach, it has been possible to keep the cell nucleation density at very high values, but at the same time, it has been possible to reduce the relative density.

The structure obtained in the second cycle compared with the one obtained in the first one presents a significant reduction of the relative density from 0.5 to 0.3 while keeping the cell size and the cell nucleation density in the same order the magnitude (Figure 2b1).

In comparison with the sample produced with similar conditions in just one cycle (31 MPa 24 °C), the introduction of a double cycle leads to a reduction of one order of magnitude in the cell size and an increase in two orders of magnitude in the cell nucleation density by almost keeping unchanged the relative density (Figure 2b2).

The achieved nanocellular material produced by the double cycle presents a unique combination of characteristics not reported up to now using a single cycle process.

**Figure 3:**
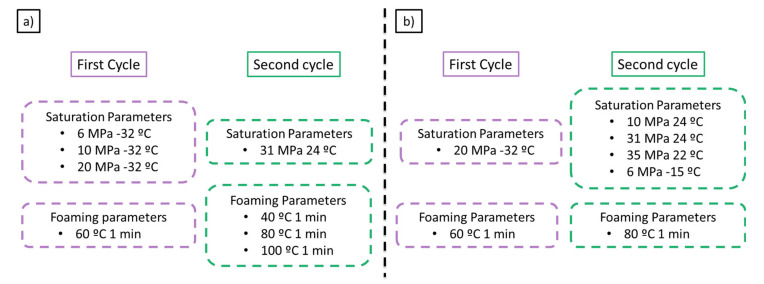
(**a**) Set of experiments to evaluate the effect of the first cycle saturation parameters and the effect of the second cycle foaming parameters on the final cellular structure. (**b**) Set of experiments carried out to evaluate the effect of the second cycle saturation parameters on the final cellular structure.

Taking into account the previous promising result a set of experiments were carried out to study the influence of the saturation and foaming parameters on the final cellular structure. Firstly, the effect of the first cycle saturation parameters was studied by changing the saturation pressure while keeping constant the saturation parameters in the second cycle at 31MPa and 24 °C (Figure 3a). Thus, saturation pressures of 6, 10, and 20 MPa at a saturation temperature of −32 °C were used in the first cycle. The foaming parameters of the second cycle have also been studied, changing the foaming temperature between 40, 80, and 100 °C with a foaming time of 1 min.

Secondly, the influence of the second cycle saturation parameters was studied while keeping constant the first cycle parameters in 20 MPa and −32 °C (Figure 3b). Saturation conditions allowing an increase in solubility in the second cycle were selected to be 10 MPa and 24 °C, 31 MPa and 24 °C, 35 MPa and 22 °C, and 6 MPa −15 °C. PMMA solubility at those conditions is 24.6 wt.%, 31.3 wt.%, 32.5 wt.%, and 34.8 wt.%, respectively.

The consequences for the cell size, cell nucleation density and relative density of all those modifications are detailed in the following sections.

### 3.2. Influence of the Parameters of the First Cycle

An increase in the saturation pressure in the first cycle leads to an increase in solubility in the material. Thus 6 MPa leads to 38.6 wt.% of CO_2_ uptake, 10 MPa to 41.0 wt.% and 20 MPa to 45.5 wt.%. As expected, smaller cell sizes and higher cell nucleation densities are obtained, as has been previously reported in the literature [15].

**Figure 4:**
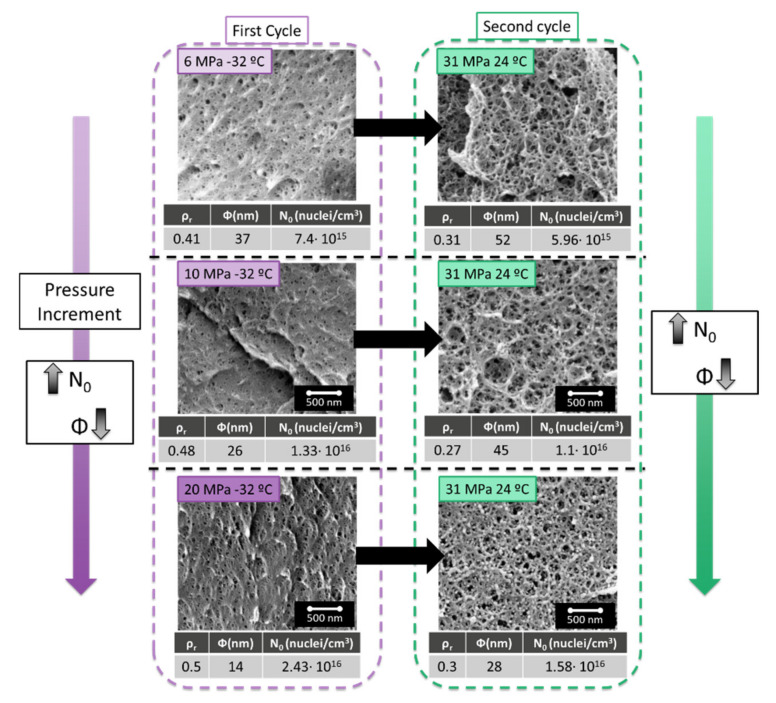
Effect of changing the first cycle parameters (6 MPa, 10 MPa, and 20 MPa and −32 °C foamed at 60 °C during 1 min) on the cellular structure obtained in the second cycle with the parameters fixed at 31 MPa and 24 °C and foamed at 80 °C 1 min.

As shown in Figure 4, the cellular structures obtained after a second cycle performed at 31 MPa and 24 °C present a smaller cell size when the cell size of the starting cellular material obtained after the first cycle is also small. The results are similar for the cell nucleation density. The higher the cell nucleation density in the first cycle, the higher is the one obtained in the second one. The materials produced present interesting characteristics with a cell density over 10^16^ cells/cm^3^ and low relative densities.

In conclusion, to minimize the cell size and maximize the cell nucleation density after the second cycle, the same should be done in the first cycle. Therefore, it is beneficial to maximize the solubility in the first cycle. On the other hand, the foaming parameters allowing a maximum cell nucleation densities in the first cycle were 60 °C and 1 min for 20 MPa and −32 °C of saturation pressure [13]. Those conditions have been therefore used in the next section of this paper.

Foaming temperatures in the second cycle have also been evaluated foaming at 40, 80, and 100 °C for 1 min. Table 1 shows this effect in samples with a fixed first cycle (20 MPa −32 °C foamed at 60 °C for 1 min) and a second cycle performed at 31 MPa 24 °C and foaming at these three temperatures for 1 min. As it can be seen relative densities were minimum through foaming at 80 °C, due to the maximization of the number of nucleation points at this temperature. Thus, for the following sections, these foaming conditions were fixed.

The results obtained in this section can be used to hypothesize about the foaming mechanisms taking place in this cycling gas dissolution foaming. As it is observed in the experimental results the cell nucleation density is almost kept when the second cycle is applied. As this second cycle has been carried out at conditions that typically provide cell nucleation densities in the range of 10^14^ cells/cm^3^, the cells in the final materials should come from the first foaming step. Therefore, we can conclude that a very high number of cells is created in the first cycle, and then the second cycle is used mainly for growth of these cells, reducing the relative density.

### 3.3. Influence of the Saturation Parameters of the Second Cycle

Considering the information extracted from the previous section, the first cycle parameters have been fixed in 20 MPa and −32 °C as saturation parameters and 60 °C and 1 min as foaming parameters. These are the conditions giving the maximum cell nucleation density.

A second cycle of gas dissolution foaming was applied to this material using four different conditions, each of them leading to a higher solubility, as previously indicated.

**Figure 5:**
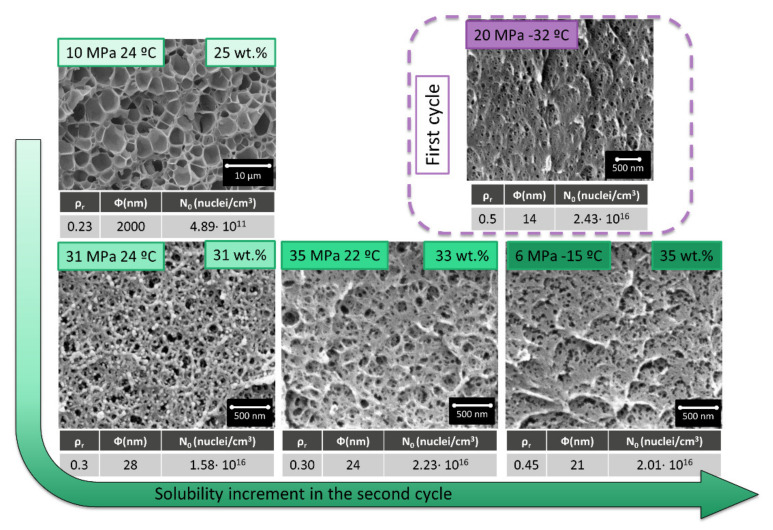
Cellular structures obtained at different saturation conditions in the second cycle (10 MPa 25 °C, 30 MPa 25 °C, 35 MPa 22 °C and 6 MPa −15 °C and foamed at 80 °C for 1 min) by fixing the saturation parameters in the first cycle at 20 MPa and −32 °C and foamed at 60 °C for 1 min.

As it can be seen in Figure 5, an increase in the solubility in the second cycle leads to a reduction in the cell size and an increase in the cell nucleation density.

**Figure 6:**
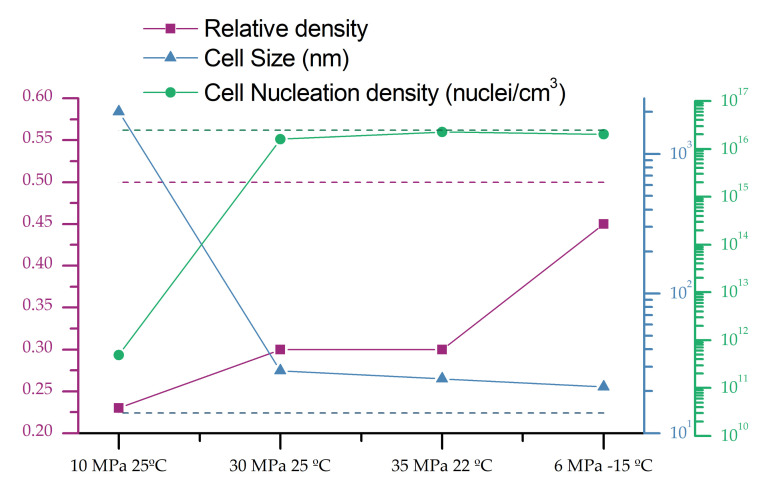
Relative density, cell size, and cell nucleation density of the samples produced with two cycles (10 MPa 25 °C, 30 MPa 25 °C, 35 MPa 22 °C and 6 MPa −15 °C and foamed at 80 °C for 1 min) by fixing the saturation parameters in the first cycle at 20 MPa and −32 °C and foamed at 60 °C for 1 min. The dotted line indicates the values obtained in a single cycle process at 20 MPa and −32 °C and foamed at 60 °C for 1 min.

Those results can be deeply analyzed by measuring the relative density, cell size, and cell nucleation density (Figure 6). The dashed line in Figure 6 indicates the values for a material produced in a single cycle at 20 MPa −32 °C, with a cell size of 14 nm, a cell nucleation density of 2.43 × 10^16^ nuclei/cm^3,^ and a relative density of 0.5. As previously commented, those cell size values are the smallest reported in the literature for PMMA, with a relative density of 0.5.

Cellular structures presented after a second cycle have a decreasing cell size and an increasing cell nucleation density as solubility rises. However, the most remarkable factor is the obtained relative density, which is always smaller than the one presented for the initial material.

Poor solubility in the second cycle of 25 wt.% (obtained at 10 MPa and 24 °C) leads to cells bigger than the micron, while the most extreme conditions of 6 MPa and −15 °C (35 wt.% of gas uptake) leads to a cellular structure very similar in density and with a larger cell size than the initial one. The most remarkable cellular structures are those obtained at intermediate saturation conditions. Saturation parameters of 30 MPa 24 °C and 35 MPa 22 °C, leads to cell sizes clearly below 50 nm and cell nucleation densities that are almost as high as that of the material obtained in the first cycle. However, the relative density of those materials is much smaller (0.3), being this combination of cellular structure parameters unique in the previous literature when only the nanocellular structure is analyzed.

The obtained results can be explained as follows: in the first cycle, a high amount of small cells are created, while in the second cycle, those cells are preferential sites to either nucleate or further grow due to the pre-existing cell decrease the energy barrier for both processes. That means in this process, it can be assumed that nucleation occurs in the first cycle and those cells can further growth in the second one if the conditions are favorable. When saturation parameters are as soft as 10 MPa 24 °C, the solubility is so small that the energy is not enough to take advantage of the previous nucleation points leading to microcellular structures. However, for both intermediate saturation conditions, 14 nm cells act as nucleation points that are further growth. When comparing the cell nucleation densities in the second and in the first cycle, 92% of the nuclei in the first cycle are able to grow in the second one. The initial 14 nm cells grow up to 24 nm leading to the observed reduction in the relative density.

Saturation conditions of 6 MPa and −15 °C lead to such high solubility that the effect is not so notable, although the mechanisms seem to remain the same. All the nucleation points are used, and slightly further growth occurs. The cell size is kept in minimal values, and the reduction in relative density is not so large.

### 3.4. Single Cycle Process vs. Double Cycle Process

In previous sections, the materials obtained with a double cycle process were compared with the ones obtained in the first cycle.

This last section is dedicated to comparing the materials obtained through a double cycle with samples produced in a single cycle process with the same production conditions of the second cycle of the double cycle samples. That means that a sample produced through a double saturation, first one at 20 MPa −32 °C and second one at 35 MPa 22 °C, will be compared with a sample produced through a single saturation at 35 MPa and 22 °C.

**Figure 7:**
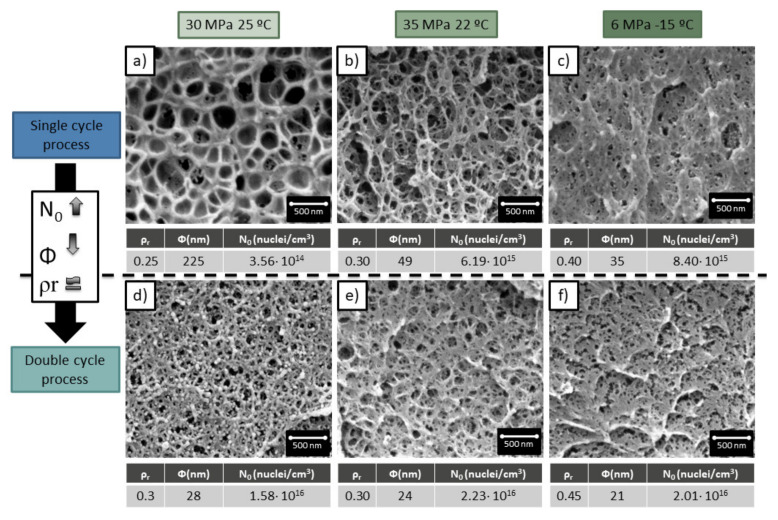
Comparison between the cellular structures obtained in a single cycle process (**a**–**c**) and a double cycle process (**d**–**f**). The single cycle process was carried out at 31 MPa 24 °C, 35 MPa 22 °C, 6 MPa −15 °C. The double cycle process is carried out at the same conditions during the first cycle and at 20 MPa −32 °C foamed at 60 °C for 1 min for the second cycle.

As is shown in Figure 7, a double cycle process results in smaller cell sizes and higher cell nucleation densities. This confirms the heterogeneous nucleation theory proposed in the previous section.

Although the cell sizes have been strongly reduced with respect to the single cycle process, the significant increment of cell nucleation density, the low relative density is almost maintained.

Therefore, it can be concluded that the newly proposed method leads to cellular materials with characteristics not achievable through the common gas dissolution process. The double cycle process allows producing cell sizes of 24 nm with relative densities of 0.3, being the minimum density reported in the literature of 0.43 for such cell sizes and cell nucleation densities of 2 × 10^16^ cells/cm^3^.

## 4. Conclusions

A double cycle gas dissolution foaming process is proposed in this work for the production of nanocellular PMMA. This process consists of a double saturation and foaming process. The PMMA is firstly saturated and foamed at certain conditions. The obtained nanocellular material is again saturated and foamed.

The influence of the production parameters has been studied. To minimize the cell size of the final material, it has been demonstrated that the cell size should also be minimum in the first cycle. A minimum cell size is reached through a maximum solubility; thus, 20 MPa and −32 °C were selected as the optimum saturation parameters in this work for the first cycle. The foaming parameters in the first cycle have been chosen to be 60 °C and 1 min to maximize the cell nucleation density.

Introducing a second cycle could lead to further growth of the cells created in the first cycle. The influence of the second cycle parameters has been studied, proving that there exists an optimum range of saturation parameters in the second cycle. Thus, a saturation pressure of 35 MPa and a saturation temperature of 22 °C leads to the production of nanocellular PMMA, combining a cell size of 24 nm with a relative density of 0.3. Those values have not been reached through a single gas dissolution foaming process for materials produced with the shape of a plate in which the skin and transition layer have been removed, herein presented for the first time.

## Figures and Tables

**Table 1:** Characteristics of nanocellular materials produced through a double cycle with different foaming temperatures in the second cycle. First cycle parameters 20 MPa −32 °C foamed at 60 °C for 1 min and second cycle parameters 31 MPa 24 °C.

Foaming Temperature (°C)	Relative Density	No (1/cm^3^)	Cell Size (nm)
40	0.49	9.22 × 10^15^	23
80	0.30	1.58 × 10^16^	28
100	0.41	1.05 × 10^16^	27
